# Sensitive Immunopeptidomics by Leveraging Available Large-Scale Multi-HLA Spectral Libraries, Data-Independent Acquisition, and MS/MS Prediction

**DOI:** 10.1016/j.mcpro.2021.100080

**Published:** 2021-04-09

**Authors:** HuiSong Pak, Justine Michaux, Florian Huber, Chloe Chong, Brian J. Stevenson, Markus Müller, George Coukos, Michal Bassani-Sternberg

**Affiliations:** 1 Department of Oncology, Ludwig Institute for Cancer Research Lausanne, Lausanne University Hospital and the University of Lausanne, Lausanne, Switzerland; 2 SIB Swiss Institute of Bioinformatics, Lausanne, Switzerland

**Keywords:** immunopeptidomics, HLA, antigen discovery, LC-MS, DDA, DIA, in silico MS/MS spectra predictions, HLA binding prediction, AGC, automatic gain control, DDA, data-dependent MS/MS acquisition, DIA, data-independent acquisition, FDR, false discovery rate, HLA, human leukocyte antigen, iRT, index retention time, MS/MS, tandem MS, PRM, parallel reaction monitoring, PSM, peptide spectrum match, RT, retention time, TAAs, tumor-associated antigens

## Abstract

Mass spectrometry (MS) is the state-of-the-art methodology for capturing the breadth and depth of the immunopeptidome across human leukocyte antigen (HLA) allotypes and cell types. The majority of studies in the immunopeptidomics field are discovery driven. Hence, data-dependent tandem MS (MS/MS) acquisition (DDA) is widely used, as it generates high-quality references of peptide fingerprints. However, DDA suffers from the stochastic selection of abundant ions that impairs sensitivity and reproducibility. In contrast, in data-independent acquisition (DIA), the systematic fragmentation and acquisition of all fragment ions within given isolation *m/z* windows yield a comprehensive map for a given sample. However, many DIA approaches commonly require generating comprehensive DDA-based spectrum libraries, which can become impractical for studying noncanonical and personalized neoantigens. Because the amount of HLA peptides eluted from biological samples such as small tissue biopsies is typically not sufficient for acquiring both meaningful DDA data necessary for generating comprehensive spectral libraries and DIA MS measurements, the implementation of DIA in the immunopeptidomics translational research domain has remained limited. We implemented a DIA immunopeptidomics workflow and assessed its sensitivity and accuracy by matching DIA data against libraries with growing complexity—from sample-specific libraries to libraries combining 2 to 40 different immunopeptidomics samples. Analyzing DIA immunopeptidomics data against a complex multi-HLA spectral library resulted in a two-fold increase in peptide identification compared with sample-specific library and in a three-fold increase compared with DDA measurements, yet with no detrimental effect on the specificity. Furthermore, we demonstrated the implementation of DIA for sensitive personalized neoantigen discovery through the analysis of DIA data with predicted MS/MS spectra of clinically relevant HLA ligands. We conclude that a comprehensive multi-HLA library for DIA approach in combination with MS/MS prediction is highly advantageous for clinical immunopeptidomics, especially when low amounts of biological samples are available.

## Highlights

• So far, DIA in the immunopeptidomics translational research has been limited.
• DIA immunopeptidomics data were analyzed against a complex multi-HLA spectral library.
• This resulted in improved sensitivity with no detrimental effect on the specificity.
• We implemented DIA for clinical antigen discovery combined with predicted MS/MS spectra.

## Graphical Abstract

**Figure:**
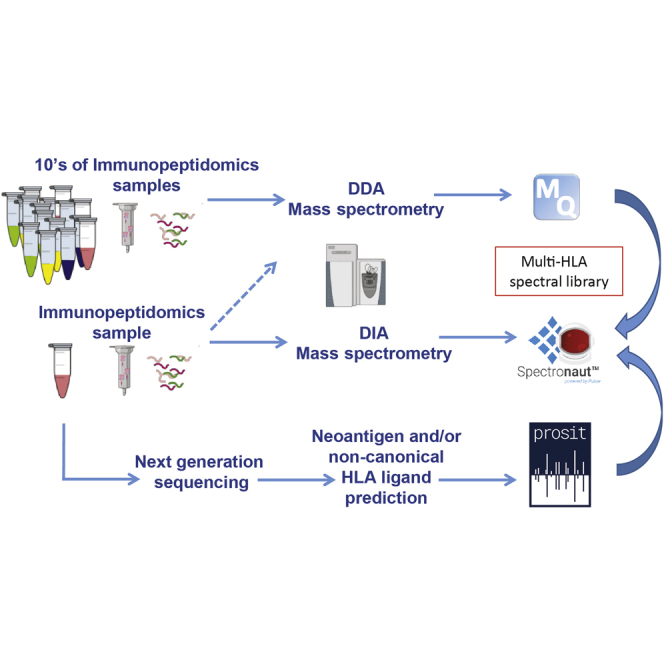

## In Brief

The implementation of DIA in the immunopeptidomics translational research domain has remained limited because the amount of HLA peptides eluted from clinical samples is typically not sufficient for acquiring both meaningful DDA data for generating comprehensive spectral libraries and DIA MS measurements. We implemented a DIA immunopeptidomics workflow and assessed its sensitivity and accuracy with libraries of growing complexity and multi-HLA libraries. In addition, we demonstrated the analysis of DIA data with predicted MS/MS spectra of clinically relevant HLA ligands.

Deep characterization of the human leukocyte antigen (HLA) immunopeptidome can advance the development of therapeutics against cancer and infectious diseases (1, 2, 3, 4). In cancer, CD8+ T cells can directly recognize and eliminate tumor cells through specific interactions between their T cell receptors and antigens presented on the tumor cells as short peptides (8–11 amino acids in length) in complex with HLA molecules. MS is the state-of-the-art methodology for capturing the breadth and depth of the immunopeptidome across HLA allotypes and cell types (5, 6). MS-based identification of cancer-specific HLA-binding peptides has led to the development of cancer vaccines and T cell–based therapies that have been shown to induce anticancer immune responses (7, 8). The likelihood of identifying clinically relevant antigens, such as mutated neoantigens (9, 10, 11, 12, 13, 14, 15) and canonical (7, 16, 17, 18) and noncanonical tumor antigens (19, 20, 21, 22), among the plethora of eluted HLA-binding peptides, increases with the depth of the ligandome. Hence, much effort has been directed at improving the sensitivity of MS-based immunopeptidomics approaches, at the peptide sample preparation step, at the MS acquisition step, and at the computational interpretation of the MS data (23, 24, 25, 26).

The majority of studies in the immunopeptidomics field are discovery driven, and data-dependent tandem MS (MS/MS) acquisition (DDA) is commonly used (6). DDA methods are ideal for the discovery of targets of interests as they generate high-quality references of peptide fingerprints, thanks to the isolation centered on the selected peptide *m/z* before MS/MS (27). However, DDA suffers from the stochastic selection of abundant ions that often leads to lower sensitivity of detected molecules and a reduced reproducibility between samples. Thus, the method is less suited to accurately quantify molecules across different samples (28, 29).

In contrast, in data-independent acquisition (DIA), the fragmentation and acquisition of all fragment ions from a predefined list of precursor isolation windows yields a comprehensive map for a given sample (30, 31). Often, either a set of small isolation windows of 2 to 5 *m/z* (32, 33) or a set of larger isolation windows of 20 *m/z* (34) is used to acquire MS/MS data and either peptide-centric (spectral library) or spectrum-centric (library-free) strategies to solve the complexity of MS/MS spectra to identify peptides. In the spectrum-centric approach, identifications are obtained after deconvolution of the acquired complex MS/MS spectra, mainly with fragmentation coelution profiles, followed by database search (35, 36, 37). In the peptide-centric strategy, *m/z* and retention time (RT) from DDA-obtained spectral libraries are used to match the DIA spectra (34, 38).

Because the amount of HLA peptides eluted from clinical samples, such as small tumor tissue biopsies, is typically not sufficient for acquiring both meaningful DDA data for generating comprehensive spectral libraries and DIA MS measurements, the implementation of DIA in the immunopeptidomics translational research domain has remained limited. Only a few proof-of-concept DIA immunopeptidomics studies in cell line models have been published to date (39, 40, 41, 42). They reported the feasibility of using large spectral libraries of peptides with defined HLA-binding specificities (39), and that, as expected, DIA outcompetes DDA in terms of both reproducibility and sensitivity when measuring low-yield samples (40, 42). For example, Caron *et al.* (6) collected high-quality immunopeptidomics MS/MS DDA data containing RT and fragmentation information for thousands of HLA ligands and generated HLA allele-specific peptide spectral libraries that are used for matching MS/MS DIA peptidomics data of HLA-matched samples. Schittenhelm *et al.* (41) combined DIA and multiple reaction monitoring to quantify HLA-B27 restricted peptides across eight of the most frequent HLA-B27 allotypes.

However, the specificity of the spectral library matching has yet to be adequately addressed especially in the context of large-scale immunopeptidomics libraries of mixed HLA restrictions (*i.e.*, multi-HLA). Furthermore, the extent to which large-scale immunopeptidomics spectral libraries across different cell types and HLA restrictions can facilitate sensitive and accurate DIA-based detection of HLA peptides has never been systematically assessed. Here, we implemented a DIA immunopeptidomics workflow and assessed its sensitivity and accuracy by matching DIA data against libraries with growing complexity—from sample-specific libraries to libraries combining 2 to 40 different immunopeptidomics samples. Analyzing DIA immunopeptidomics data against a multi-HLA spectral library resulted in a two-fold increase in peptide identification compared with a sample-specific library and a three-fold increase compared with DDA measurements, yet with no detrimental effect on the specificity. Furthermore, we demonstrated how integration of Prosit-based prediction of MS/MS spectra with DIA-based immunopeptidomics can lead to the sensitive detection of neoantigens and other clinically relevant antigens.

## Experimental Procedures

### Experimental Design and Statistical Rationale

We collected HLA-I peptidomics datasets for re-analysis from the following ProteomeXchange (43) sources: PXD000394, PXD013649, PXD013831, PXD006939, PXD014017, and PXD009925 (20, 24, 44, 45, 46, 47, 48). In addition, we included new DDA immunopeptidomics datasets for the B cell lines RA957 and JY, and a T cell sample TIL1. We generated 2 to 3 replicates of DIA measurements of immunopeptidomics samples from RA957, JY, and OD5P samples, without inclusion of RT internal standards. Information about the number and type of samples and the name of raw files and their inclusion in different spectral libraries is available in supplemental Table S1. In total, we included 41 biological samples (cell lines or tissues), each measured across different technical and/or biological replicates, reaching in total 217 raw files. This collection of samples covered 23 HLA-A, 27 HLA-B, and 21 HLA-C alleles. HLA typing information for all samples included in the article is provided in supplemental Table S2. This work abides by the Declaration of Helsinki principles, and the translational research has been approved by the Centre Hospitalier Universitaire Vaudois ethics committee (protocols 2017-00305).

### Purification of HLA-Binding Peptides

We performed HLA immunoaffinity purification according to our previously established protocols (24, 49). W6/32 mAbs were purified from the supernatants of HB95 (ATCC HB-95) using protein-A Sepharose 4B (Pro-A) beads (Invitrogen), and antibodies were then cross-linked to Pro-A beads. Cells were lysed with PBS containing 0.25% sodium deoxycholate (Sigma-Aldrich), 0.2 mM iodoacetamide (Sigma-Aldrich), 1 mM EDTA, a 1:200 protease inhibitor cocktail (Sigma-Aldrich), 1 mM phenylmethylsulfonyl fluoride (Roche), and 1% octyl-beta-D glucopyranoside (Sigma-Aldrich) at 4 °C for 1 h. The lysates were cleared by centrifugation in a table-top centrifuge (Eppendorf) at 4 °C for 50 min at 21,191*g*. We used the Waters Positive Pressure-96 Processor (Waters) and 96-well single-use microplates with 3-μm glass fibers and 10-μm polypropylene membranes (Seahorse Bioscience, ref no: 360063). The lysates were passed through a plate containing pan HLA-I antibody-crosslinked beads at 4 °C. The beads in the plates were then washed with varying concentrations of salts using the processor. Finally, the beads were washed twice with 2 ml of 20 mM Tris HCl, pH 8. Sep-Pak tC18 100-mg Sorbent 96-well plates (Waters, ref no: 186002321) were used for the purification and concentration of HLA-I peptides. The C18 sorbents were conditioned, and the HLA complexes and bound peptides were directly eluted from the affinity plate with 1% TFA (Sigma-Aldrich). After washing the C18 sorbents with 2-ml of 0.1% TFA, HLA-I peptides were eluted with 28% acetonitrile (Sigma-Aldrich) in 0.1% TFA. Recovered HLA-I peptides were dried using vacuum centrifugation (Concentrator plus, Eppendorf) and stored at −20 °C.

### LC-MS/MS DDA and DIA Analyses

The LC-MS/MS system consisted of an Easy-nLC 1200 (Thermo Fisher Scientific) hyphenated to a Q Exactive HF-X mass spectrometer (Thermo Fisher Scientific). Peptides were separated on a 450-mm analytical column (8-μm tip, 75-μm inner diameter, PicoTip emitter, New Objective) packed with ReproSil-Pur C18 (1.9-μm particles, 120 Å pore size, Dr Maisch GmbH). The separation was performed at a flow rate of 250 nl/min by a gradient of 0.1% formic acid in 80% acetonitrile (solvent B) and 0.1% formic acid in water (solvent A). HLA-I peptides were analyzed by the following gradient: 0 to 5 min (5% B); 5 to 85 min (5–35% B); 85 to 100 min (35–60% B); 100 to 105 min (60–95% B); 105 to 110 min (95% B); 110 to 115 min (95–2% B); and 115 to 125 min (2% B).

Two different parameter settings were used to acquire DDA data for the sample-specific libraries (recent data) and the combined, mixed, and BigLib libraries that composed of old data. For DDA measurements, full MS spectra were acquired in the Orbitrap from *m/z* = 300 to 1650 with a resolution of 60,000 (*m/z* = 200) and an ion-accumulation time of 80 ms. The automatic gain control (AGC) was set to 3e6 ions. MS/MS spectra were acquired in a data-dependent manner on the 20 most abundant precursor ions with a resolution of 30,000 (*m/z* = 200) for sample-specific and on the ten most abundant precursor ions with a resolution of 15,000 (*m/z* = 200) for the other libraries. The ion-accumulation time was set to 120 ms with an isolation window of 1.2 *m/z*. The AGC was set to 2e5 ions, the dynamic exclusion was set to 20 s, and a normalized collision energy of 27 was used for fragmentation. No fragmentation was performed for HLA-I peptides with assigned precursor ion charge states of four and above, and the peptide match option was disabled.

For DIA measurements, the cycle of acquisitions consists of a full MS scan from 300 to 1650 *m/z* (R = 60,000 and ion accumulation time of 60 ms) and 22 DIA MS/MS scans in the orbitrap (supplemental Table S3). For each DIA MS/MS scan, a resolution of 30,000, an AGC of 3e6, and a stepped normalized collision energy (25.5, 27, and 30) were used. The maximum ion accumulation was set to auto, the fixed first mass was set to 200 *m/z*, and the overlap between consecutive MS/MS scans was 1 *m/z*.

### DDA and DIA Data Analyses

We used the MaxQuant computational platform, version 1.5.5.1 (50), to search the peak lists against the UniProt databases (human 42,148 entries, March 2017) and a file containing 246 frequently observed contaminants. The default settings were used except the following parameters: enzyme specificity was set to ‘unspecific,’ methionine oxidation and protein N-term acetylation were set as variable modifications and no fixed modification was set, and peptide spectrum match (PSM) false discovery rate (FDR) was set to 0.01 with no protein FDR. The initial allowed mass deviation of the precursor ion was set to 6 ppm, and the maximum fragment mass deviation was set to 20 ppm.

The spectral library for each sample was generated using results from MaxQuant discovery search for each experiment (sample-specific, combined, mixed, and BigLib) by uploading mqpar.xml, msms.txt, evidence.txt, and fasta files into Spectronaut (version 14.6.2, Biognosys). The default settings were used except the following parameters. For the mass tolerance, the calibration search was set to ‘dynamic,’ and a correction factor of 1 was used for MS and MS/MS. For identification, an FDR threshold of 0.01 and unspecific digestion rule were used in agreement with the MaxQuant search. For spectral library filtering, we applied the default settings, meaning that the minimum fragment length was set to 3 and only peptide identifications with at least three best fragments (up to 6) were considered. A deep learning–assisted index retention time (iRT) regression and an R-square of 0.8 were used for iRT reference strategy and correlation score, respectively. The selection of fragment ions based on intensity was used. For identification, the library was matched against JY, OD5P and RA957 DIA MS/MS data with q-value cut-off of 0.01 and 1, respectively, for precursor and protein (51). MS and MS/MS data were extracted using maximum intensity strategy within the given mass tolerance. A dynamic mass tolerance and a correction factor of 1 were used for both MS and MS/MS. The quantification was done at MS/MS level, using at least three fragments. A global normalization per run based on the median was used for cross run normalization.

The results from Spectronaut were exported in MSstat file and two custom peptide-centered file formats: peptide quantity and peptide score. In peptide quantity export, columns corresponding to PG.Genes, PG.UNiProtIds, PG.ProteinNames, PEP.StrippedSequence, EG.PrecursorId, EG.ModifiedSequence, PEP.Quantity, EG.Qvalue, and EG.ApexRT were exported to analyze data from sample-specific to BigLib library (supplemental Tables S4–S8). From this export, reverse hits and contaminant peptides were removed in agreement with MaxQuant search before data analysis. For peptide score export, columns corresponding to R.FileName, PEP.GroupingKey, PEP.Quantity, EG.iRTPredicted, EG.PrecursorId, EG.Pvalue, EG.Qvalue, EG.ApexRT, EG.Cscore, EG.IntCorrScore, FG.FragmentCount, and FG.ShapeQualityScore were exported to analyze predicted peptides (supplemental Table S9).

### HLA-I-Binding Predictions and Peptide Clustering

To evaluate the binding affinity of identified peptides to the respective HLA-I molecules, NetMHCpan 4.1 prediction software (52) was run on all identified peptides ranging in length from 9 to 13 amino acids. Peptides with a rank of ≤2% were considered as binders. For each peptide, the HLA allele with the lowest rank was assigned. Binding motif deconvolution of 9 mer HLA-I peptides was performed using the MixMHCp 2.1 (47, 53), with the default settings except for the number of maximum motifs set to 10 motifs. Upon completion of deconvolution, motifs were manually analyzed and assigned to patient HLA allotypes.

### DIA Data Analysis With *In Silico*–Predicted MS/MS

Spectrum predictions were performed by a locally installed docker version of the Prosit software (54), version 1.1, obtained from https://github.com/kusterlab/prosit. The nontryptic HCD fragmentation model (Schmidt, Tobias; Wilhelm, Mathias (2020): https://doi.org/10.6084/m9.figshare.12936947.v1) and the iRT model (Schmidt, Tobias (2018): https://doi.org/10.6084/m9.figshare.6965801.v1) were downloaded from https://figshare.com/projects/prosit/35582. Our installation of Prosit ran on a NVIDIA GeForce RTX 2080 Ti graphic card with 11 GB GDDR6 memory and required 6 ms to predict a single spectrum, where the computation time scaled linearly with the number of spectra. If not otherwise indicated, Prosit predicted a spectrum of charge 1, 2, and 3 for each peptide. Methionine oxidation M(ox) was used as a variable modification, that is, for a methionine containing peptide, all combinations of M and M(ox) were used with up to 2 oxidations per peptide. Cysteine-containing peptides were excluded from this study because Prosit assumes all cysteines are carbamidomethylated, which is not the case for our sample preparation. After Prosit prediction, spectra were annotated with a protein ID for compatibility with Spectronaut.

Spectral libraries were predicted by Prosit as mentioned above for all peptides included in the BigLib library (DDA-to-Prosit-to-DIA) and analyzed by Spectronaut using the same settings described above. A list of 39 peptides derived from known tumor-associated antigens (TAAs) identified in OD5P sample with the DDA method (1% FDR) and validated with parallel reaction monitoring (PRM) was obtained from Chong *et al.* (20). We compared their detection in the OD5P DIA data analyzed against the BigLib (DDA-to-DIA) and compared their detection using Prosit-predicted BigLib library (DDA-to-Prosit-to-DIA).

In addition, for the detection of peptides derived from the translation product of the novel ORF in the ABCB5 gene previously reported by Chong *et al.* (20), we performed MaxQuant analysis of the 16 OD5P DDA measurements reported previously by Chong *et al.* We searched the MS/MS data against the UniProt databases (human 42,148 entries, March 2017) in which the in silico–translated product of the novel ORF of ABCB5 was included (DDA-to-DIA). Here, a PSM FDR of 3% was set in MaxQuant to be in agreement with the discovery search done by Chong *et al.* (20). In addition, to explore if additional predicted peptides from the ABCB5 novel ORF could be identified in the DIA analysis, even if not detected initially by DDA, a generic list of 9- and 10-mer HLA-I peptides predicted to bind frequent HLA allotypes (A∗01:01, A∗02:01, A∗03:01, A∗24:02, A∗26:01, B∗07:02, B∗08:01, B∗27:05, B∗39:01, B∗40:01, B∗58:01, B∗15:01) was generated with the PRIME (https://github.com/GfellerLab/PRIME) algorithm (55) (rank ≤ 1%; 27 peptide candidates; supplemental Table S10). 17 non–cysteine-containing noncanonical peptide candidates were then predicted by Prosit (Prosit-to-DIA) with the settings described above.

Prosit-generated outputs were analyzed against OD5P DIA data with Spectronaut using the settings described above with a precursor q-value cut-off of 0.01. To estimate the quality of matched TAAs and ABCB5-derived peptides with the predicted libraries, we extracted each matched feature with elution group scores (EG.Cscore), the Spectronaut identification score, which is based on advanced mProphet scoring where a high score indicates high-quality identification (56).

## Results

### Sensitivity of DIA Immunopeptidomics With Sample-Specific DDA Libraries

**Fig. 1:**
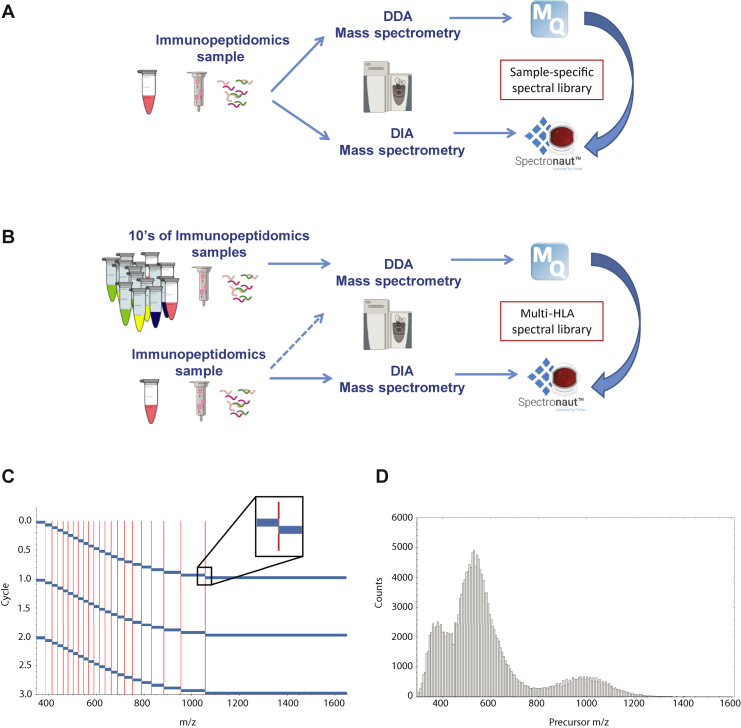
**Overview of the application of DIA immunopeptidomics.** A schematic overview of the application of sample-specific library for DIA approach for immunopeptidomics (*A*) and multi-HLA library for DIA immunopeptidomics leveraging existing large-scale immunopeptidomics datasets (*B*). As a proof of concept, we applied the MaxQuant and Spectronaut computational environments for DDA library preparation and DIA data analysis, respectively. Schematic representation of the variable isolation windows across the *m/z* space applied for the DIA acquisition method (*C*). Histogram of *m/z* values of all HLA peptides included in the multi-HLA BigLib spectral library (*D*). DDA, data-dependent MS/MS acquisition; DIA, data-independent acquisition; HLA, human leukocyte antigen.

We started our investigation of assessing the sensitivity of a DIA immunopeptidomics approach using sample-specific spectral libraries (Fig. 1*A*) to reflect the ‘best case’ results that would then allow comparisons with multi-HLA spectral libraries generated from publicly available DDA data from our laboratory (Fig. 1*B*). We applied overall standard DIA parameters (see the Experimental Procedures section for more details). However, because we routinely select single-, double-, and triple-charged ions, we extended the *m/z* range to 1650 and set isolation windows with varying sizes along the *m/z* scale (Fig. 1, *C* and *D* and supplemental Table S3).

**Fig. 2:**
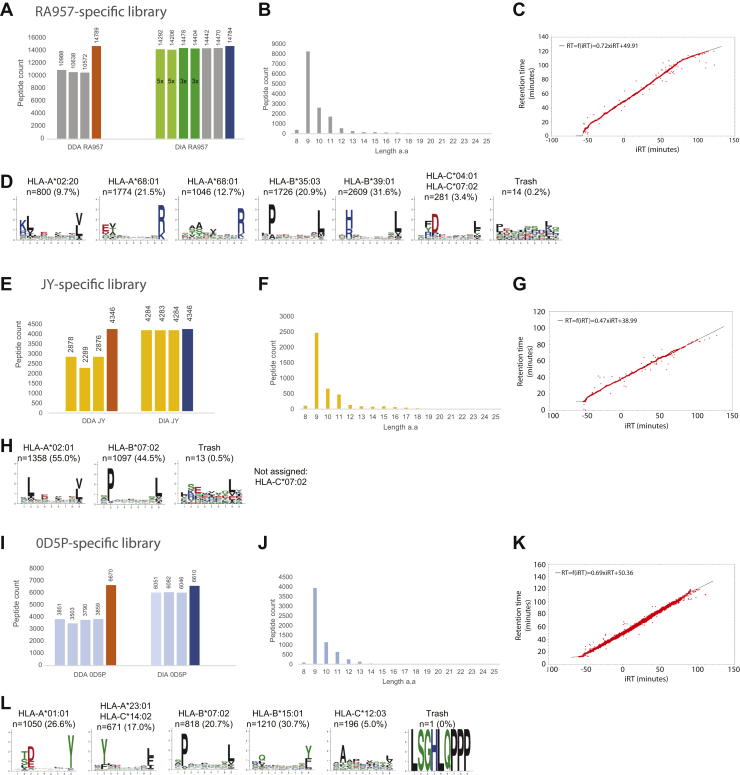
**Application of sample-specific spectral libraries for matching immunopeptidomics DIA data.** HLA-I peptides purified from RA957 cells were measured by DIA in duplicates as normal and 3× and 5× diluted samples and matched against a library constructed from three DDA replicates, all measured sequentially (*A*). The number of peptides identified in each of the DDA measurements included in the RA957-specific library and the number of peptides identified in each of the DIA measurements following matching to this library are reported. The *orange bar* refers to the number of peptides identified in three DDA runs, and the *blue bar* refers to the total number of peptides included in the DIA library. Length distribution of peptides identified in RA957 DIA samples (*B*). Representative correlation between measured RT in the DIA data and the predicted RT calculated by Spectronaut (*C*). Deconvolution of the consensus binding motifs of RA957 DIA immunopeptidomics samples (*D*). The number of peptides and the HLA restriction assigned to each motif are reported. Two subspecificities were detected for HLA-A∗68:01. HLA-I peptide samples purified from JY cells were measured by DIA in triplicates and matched against a JY-specific library constructed from three DDA replicates (*E*). The length distribution of peptides identified in JY DIA samples (*F*) and a representative correlation between measured RT and the predicted RT (*G*) are provided. Deconvolution of the consensus binding motifs of JY DIA immunopeptidomics samples (*H*). HLA-I peptide samples purified from OD5P cells were measured by DIA in triplicates and matched against a OD5P-specific library constructed from four DDA replicates (*I*). The length distribution of peptides identified in OD5P DIA samples (*J*) and a representative correlation between measured RT and the predicted RT (*K*) are provided. Deconvolution of the consensus binding motifs of OD5P DIA immunopeptidomics samples (*L*). DDA, data-dependent MS/MS acquisition; DIA, data-independent acquisition; HLA, human leukocyte antigen; RT, retention time.

We isolated from the RA957B cell line HLA-I-binding peptides by immunoaffinity purification with the pan-HLA W6/32 antibody followed by a desalting step. We measured three technical replicates of this HLA-I immunopeptidome sample by DDA. With the same instrument and LC-MS setup, we measured two additional DIA measurements of the same RA957 immunopeptidome sample, as well as two technical replicates, each of the sample diluted 3-fold (3×) and 5-fold (5×) (supplemental Table S1). We searched the DDA MS/MS data with MaxQuant at 1% FDR and identified in the three replicates 10,988, 10,638, and 10,572 peptides, respectively, and 14,789 unique peptides in total. With Spectronaut, we generated a spectral library, comprising in total 14,784 unique peptide identifications. We matched the RA957 immunopeptidomics DIA data against this RA957-specific library (q-value ≤0.01) and identified in two DIA measurements 14,442 and 14,470 unique peptides, respectively, recovering more than 97% of the peptides included in the library (Fig. 2*A* and supplemental Table S4). In addition, we assessed the overall accuracy of the HLA-I peptide identification by (1) observing peptide length distribution, (2) the correlations between measured RT in the DIA data and the predicted RT calculated by Spectronaut, (3) an unbiased approach to reveal the consensus HLA-I-binding motifs with the MixMHCpred tool (47, 53), and (4) calculating the fraction of peptides predicted by NetMHCpan (52) to bind the expressed HLA allotypes (rank ≤ 2%) (supplemental Table S2). The identified peptides recapitulated the expected length distribution (an average length of 9.96 amino acids), and RTs were well correlated (Fig. 2, *B* and *C*). In addition, clustering of the 9 mer peptides with MixMHCp tool revealed the typical binding-motif specificities of the HLA allotypes expressed in the RA957 sample (Fig. 2*D*), which were highly similar to the reference motifs from NetMHCpan motif viewer (supplemental Fig. S1). Two subspecificities were detected for HLA-A∗68:01 as reported previously (47). The only observed exception was related to the HLA-C∗04:01 and HLA-C∗07:02 allotypes, where the relatively low number of identified peptides (n = 281 of 9-mers) matching these allotypes clustered into a single motif. Overall, 96% of the peptides (of length 9–13 amino acids) were predicted to bind the respective HLA allotypes. The overlap between the three DDA replicate samples was 66% compared with 99% for the two normal DIA replicates. Even in the 3× and 5× diluted RA957 samples (supplemental Fig. S2, *A* and *B*), a similar depth was achieved, with 14,441 and 14,395 unique sequences identified on average, respectively (Fig. 2*A* and supplemental Table S4). The quality of the peptide identifications, in terms of the peptide length and binding specificity, was similarly high. As expected, the reproducibility of peptide detection and quantification in the DIA measurements was higher than in the DDA; averaged Pearson correlations of r = 0.97 and 0.87 were calculated for comparing the pairs of DIA replicates and the three DDA replicates, respectively (supplemental Fig. S2, *C* and *D*).

Next, we compared the sensitivity of matching DIA MS measurements against a sample-specific spectral library created from DDA data measured several months apart in our laboratory, with a similar Orbitrap MS instrument, acquisition parameters, and liquid chromatography conditions. We created a JY-specific spectral library comprising three previously available MS measurements of the HLA-I peptidome of the B cell line JY (supplemental Table S1). We purified HLA peptides from a pellet of cells that was a replica of the sample used for the DDA measurements, and we performed three DIA MS measurements. Overall, 4284 peptides were identified that recapitulated the expected length distribution (an average length of 10.15 amino acids) that is typical for the HLA allotypes expressed in the JY sample and RTs were well correlated (Fig. 2, *F* and *G* and supplemental Table S4). 95% of the peptides identified with the JY sample–specific library were predicted to bind the HLA allotypes expressed in JY cells, and the resolved binding motifs were found, as expected, to be specific for the HLA-A∗02:01 and HLA-B∗07:02 allotypes (Fig. 2*H*). Additional motif for HLA-C∗07:02 could not be resolved, likely because of the lower expression of the HLA-C molecule and the relatively lower contribution of HLA-C–binding peptides to the peptidome of JY cells.

We further explored the performance of this approach by matching DIA measurements of HLA-I-binding peptides against sample-specific library of immunopeptidomics data generated in our laboratory, again with a similar Orbitrap MS instrument, acquisition parameters, and liquid chromatography conditions, however, this time with peptides eluted from biological replicates of the OD5P melanoma cells. We created a OD5P -specific spectral library containing 6610 peptide sequences identified in four independent biological replicates of peptides eluted from the OD5P cells treated or not with decitabine (two replicates each) (20) (supplemental Table S1). We generated a new HLA-I peptidomic sample from the OD5P melanoma cell line and measured three replicates with the DIA method. We matched these data against the OD5P-specific library and identified on average 6060 peptides in the DIA replicates, compared with 3503 to 3859 peptides in the four DDA measurements (Fig. 2*I* and supplemental Table S4). 98% of the peptides were predicted to bind the HLA allotypes expressed in OD5P, and the peptides similarly revealed the expected length distribution (an average length of 9.6 amino acids) and binding motifs and RTs were well correlated (Fig. 2, *J*–*L*). Here, as the binding motifs of HLA-A∗23:01 and HLA-C∗14:02 are highly similar (supplemental Fig. S1), only one motif was defined to capture this specificity. We conclude from these initial analyses that as expected, using sample-specific libraries, the DIA approach outperforms the DDA approach in terms of peptide coverage and reproducibility, and because only relevant peptides are included in such sample-specific libraries, the identifications are as accurate as in the DDA approach.

### Specificity and Sensitivity of DIA Immunopeptidomics Using Combined Spectral Libraries From Two or More Samples With Shared HLA Allotypes and Binding Motifs

Often, the low quantity of eluted HLA-I peptides from a given sample limits the number of MS measurements that can be performed, and hence, sample-specific libraries could have a limited coverage. However, common peptides may be detected in the immunopeptidome of different biological samples that share one or more HLA-I alleles or that express HLA-I alleles with similar binding motifs. We envisioned that a DIA immunopeptidomics approach would benefit from spectral libraries generated across samples; however, there could be a risk of false matches. To test the specificity of such an approach, we created different spectral libraries with growing complexity and systematically assessed peptide coverage and the fraction of predicted binders. First, we generated three different combined libraries: JY+OD5P (10,367 peptides), RA957+JY (15,456 peptides), and OD5P+RA957 (21,328 peptides) (supplemental Table S1). The DIA measurements of HLA-I peptides from JY and from OD5P samples were matched against the combined JY+OD5P library. JY and OD5P cells share the HLA-B∗07:02 allele, and we observed a small increase of around 300 to 500 identified peptides in JY and OD5P samples, respectively (supplemental Fig. S3*A* and supplemental Table S5). The average peptide length of 10.07 and 9.67 amino acids were observed for JY and OD5P samples, respectively. Importantly, although the binding motifs of the remaining HLA allotypes expressed in these cells are different and hence most of the peptides included in the JY+OD5P library are unique to one of the samples, we confirmed that no apparent noise was introduced when applying this combined library. The fraction of predicted binders remained high for JY and OD5P samples, 95% and 98%, respectively, and the binding motifs remained similar to those obtained when using the sample-specific libraries (supplemental Fig. S3, *B* and *C*).

**Fig. 3:**
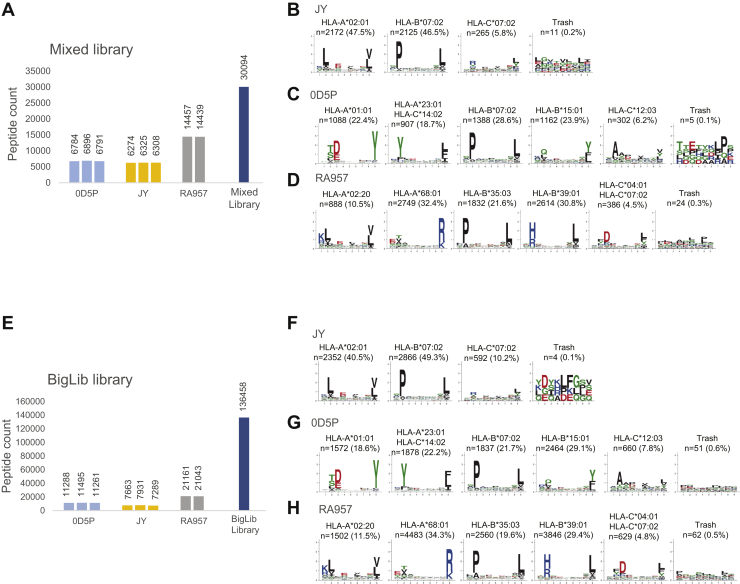
**Application of mixed and BigLib multi-HLA spectral libraries for matching immunopeptidomics DIA data.** DIA data of JY, OD5P, and RA957 samples were matched against the mixed library comprising immunopeptidomics data from six different biological samples (*A*). The number of peptides identified in each of the DIA measurements and the number of peptides included in the mixed library are reported. Deconvolution of the consensus binding motifs of JY (*B*), OD5P (*C*), and RA957 (*D*) DIA immunopeptidomics samples with MixMHCp 2.1 and manual annotation of motifs. The number of peptides and the HLA restriction assigned to each motif are reported. DIA data of JY, OD5P, and RA957 samples were matched against the BigLib library comprising immunopeptidomics data from 40 different biological samples (*E*). The number of peptides identified in each of the DIA measurements and the number of peptides included in the mixed library are reported. Deconvolution of the consensus binding motifs of JY (*F*), OD5P (*G*), and RA957 (*H*) DIA immunopeptidomics samples as described above. DIA, data-independent acquisition; HLA, human leukocyte antigen.

JY and RA957 cells share the HLA-C∗07:02 allele, and the motifs of HLA-B∗07:02 and HLA-B∗35:03 and of HLA-A∗02:02 and HLA-A∗02:20 are highly similar. We similarly found an increase of about 400 peptides in the JY DIA samples when matched against the JY+RA957 library and the clustering of all the 9-mer peptides revealed a third motif corresponding to the HLA-C∗07:02 allotype, comprising 5.5% of the peptides (supplemental Fig. S3, *D* and *E* and supplemental Table S5). Importantly, although the JY+RA957 library contained more than 3-fold the number of peptides originating from the RA957 DDA samples, no distinct motifs matching the RA957-specific allotypes (HLA-A∗68:01, HLA-B∗39:01, or HLA-C∗C:04:01) were observed in the unbiased clustering of the peptides identified in JY DIA samples (Fig. 3*E*), suggesting a minimal rate of false matching with the combined library. Overall, the percentage of binders was as high as with the analysis done with JY-specific library, with 94% of the peptides predicted as binders (an average length of 9.98). No significant increase in the number of identified peptides in RA957 DIA data was found by matching to the JY+RA957 library (supplemental Fig. S3*D*). However, we confirmed that the binding motifs remained the same and still 96% of the peptides were predicted as binders (an average length of 9.97) (supplemental Fig. S3*F*).

Interestingly, by matching DIA data of OD5P against the OD5P+RA957 library, we observed a decrease in detection of around 500 peptides, and again no difference in the number of identified peptides in the RA957 DIA data (supplemental Fig. S3*G* and supplemental Table S5). The fact that no common HLA alleles are expressed in these 2 cell lines, and that the only commonality stems from the similar motifs of HLA-B∗07:02 and HLA-B∗35:03, could potentially explain this outcome. In addition, FDR calculations applied to different combinations of samples could slightly alter the peptide yields and the repertoire. Nevertheless, the high accuracy of this analysis is demonstrated by the high percentage of peptides predicted as binders in both cell lines, 97% in both OD5P and RA957, the consistent average peptide length of 9.62 and 9.97, in OD5P and RA957, respectively, and by the resulting binding motifs that are identical to those obtained with sample-specific libraries (supplemental Fig. S3, *H* and *I*). We conclude that a DIA immunopeptidomics approach can indeed benefit from spectral libraries generated from two different samples when HLA allotypes characterized with similar binding motifs are expressed; yet, there is a risk of lower detection level when there is little overlap.

### Enhanced Sensitivity of DIA Immunopeptidomics in a Multi-HLA Library Improves the Identification of Tumor Antigens

To further explore the specificity and sensitivity of matching DIA immunopeptidomics data against a complex library, we generated a mixed library by including in total 12 DDA measurements of HLA-I peptides eluted from six samples, JY, RA957, OD5P, the melanoma cell lines ME290 and ME275, and the T cells sample TIL1 (supplemental Table S1). Each of these samples share from one to three HLA-I alleles or binding motifs (supplemental Table S2). The DIA measurements of RA957, JY, and OD5P were matched against the 30,094 peptide sequences included in this mixed library. Overall, the number of peptides identified in JY and OD5P samples further increased, reaching on average 6302 and 6824 peptides (equivalent to 6740 and 7430 in total, respectively) (Fig. 3*A* and supplemental Table S6). Only a modest increase in RA957 samples was observed, where on average 14,448 peptides (14,820 peptides in total) were identified (Fig. 3*A*). We found that the specificity remained high, as 96%, 94%, and 96% of the peptides were predicted as binders and the calculated average peptide length was 9.98, 9.93, and 9.67 amino acids in the RA957, JY, and OD5P samples, respectively. The binding motifs were similar to those obtained with sample-specific libraries, with the exception again of the detection of a third motif corresponding to the HLA-C∗07:02 in the JY peptidome comprising 5.8% of the 9 mer peptides (Fig. 3, *B*–*D*).

**Fig. 4:**
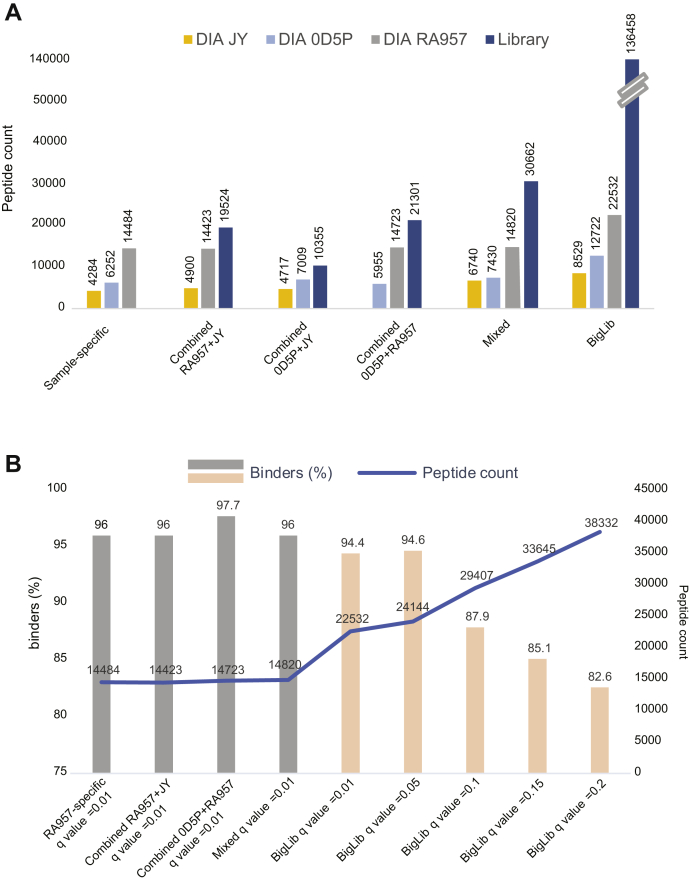
**A summary overview of peptides identified across the different analyses.** A summary overview of the number of peptides identified by matching the DIA of RA957, JY, and OD5P immunopeptidomics data against each of the sample-specific, combined, mixed, and BigLib libraries (*A*). A summary overview of the fraction of peptides predicted as binders in RA957 DIA samples matched against the RA957-specific, RA957+JY, OD5P+RA957, mixed, and BigLib libraries applying q values of 0.01 and against the BigLib library applying q values of 0.05, 0.1, 0.15, and 0.2, respectively (*B*). The total number of identified peptides in each of the abovementioned assays is reported. DIA, data-independent acquisition.

Finally, we matched the DIA against a larger multi-HLA library, called BigLib, comprising 136,458 peptide sequences identified in 190 publicly available measurements of eluted HLA-binding peptides in 40 samples from multiple and diverse biological sources, including B cells (not including JY cells), T cells, colon cancer organoids, meningioma tissues, and melanoma cell lines treated or not with interferon gamma or decitabine, covering 23 HLA-A, 27 HLA-B, and 21 HLA-C alleles. The samples were measured in our laboratory in the last 4 years, using similar LC-MS settings (supplemental Table S1). Interestingly, even when we matched JY DIA data against the BigLib library that lacks DDA data of JY cells, 8529 peptides in total were identified (Fig. 3*E* and supplemental Table S7). JY cells express HLA allotypes that are among the most highly frequent in humans and therefore are highly represented in the BigLib library (supplemental Table S2). In addition to obtaining clear motifs of the HLA-A∗02:01 and HLA-B∗07:02 alleles, we found an increase in the number of peptides fitting the HLA-C∗07:02 motif (10.2% of the 9 mer peptides) (Fig. 3*F*). We also observed a substantial increase in peptide identification in both OD5P and RA957 samples, where in total 12,722 and 22,532 unique peptides were identified, respectively (Fig. 3*E* and supplemental Table S7), which clustered to reveal the expected binding motifs (Fig. 3, *G* and *H*). Applying the multi-HLA BigLib library resulted in 1.5-fold (RA957) and 2-fold (JY and OD5P) increase in peptide coverage, compared with that obtained with the sample-specific libraries and a 2.1-, 3.2-, and 3.4-fold increase (RA957, JY, and OD5P, respectively) compared with the DDA measurements (Fig. 4*A*). The additional peptides fitted the binding motifs of each of the expressed HLA allotypes expressed in JY, OD5P, and RA957 samples, and the specificity remained high, with 92%, 95.7%, and 94% of peptides predicted as binders with average peptide lengths of 9.78, 9.58, and 9.93 amino acids, respectively.

To better assess the level of error that could potentially result from matching DIA immunopeptidomics data against a complex multi-HLA library, we matched the RA957 DIA data against the BigLib, applying higher precursor q-value thresholds, including q-values of 0.01, 0.05, 0.1, 0.15, and 0.2. We compared peptide yields and the fraction of peptides that are predicted as binders also with the results obtained above with RA957-specific, combined RA957+JY, combined OD5P+RA957, and the mixed libraries. Overall, and as expected, with the application of higher q-values, the coverage of peptide identification increased and the accuracy decreased, considering EG.Cscore distributions and delta RT values (Fig. 4*B* and supplemental Fig. S4). A drop in the fraction of peptides predicted as binders with q-values equal to 0.1 was found, where less than 87.9% of the peptides were predicted as binders. In addition, with q-values of 0.01, the fraction of binders obtained with the BigLib library was similar to the fraction obtained with the RA957-specific, combined RA957+JY, combined OD5P+RA957, and mixed libraries, in the range of 94.4 to 97.7%. Overall, with the BigLib and precursor q-value threshold of 0.01, we improved the detection rate of HLA peptides in all three investigated samples without negatively affecting the accuracy.

### Identification of Clinically Relevant Antigens by Analyzing DIA Immunopeptidomics Data With Predicted MS/MS Spectra

**Fig. 5:**
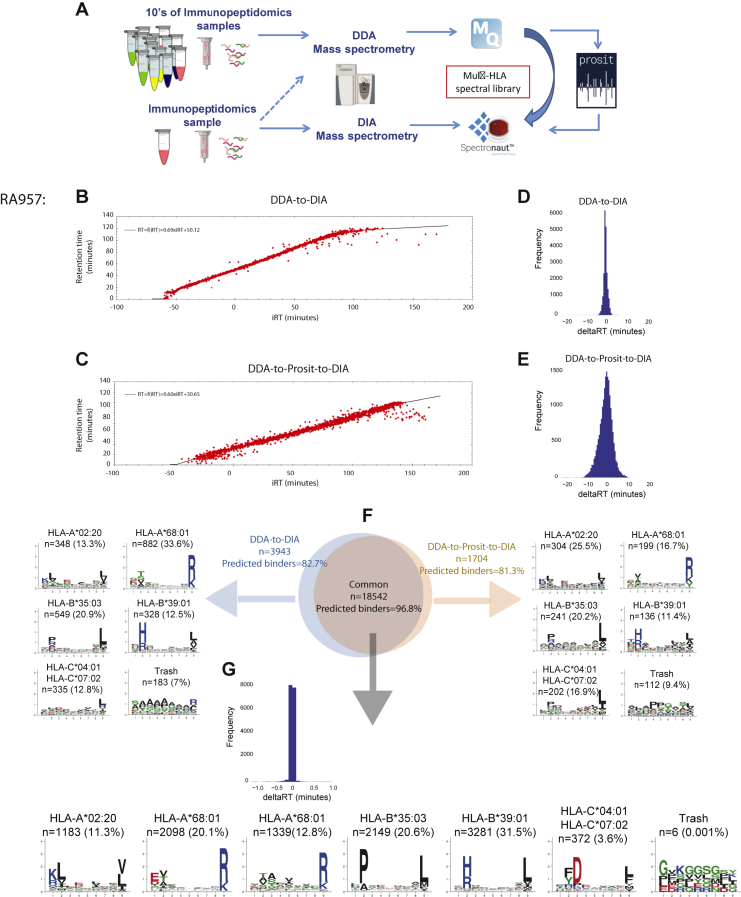
**Overview of the performance of Prosit-predicted MS/MS spectral library.** A schematic overview of our approach to estimate the performance of Prosit-predicted MS/MS spectral library of all HLA peptides included in the BigLib that were used to match DIA data of RA957 samples (*A*). Correlations between measured RT and the predicted RT calculated by Spectronaut (RT = f(iRT)) of peptides identified in RA957 applying the BigLib library in the DDA-to-DIA approach (*B*) and with the Prosit-predicted BigLib library in the DDA-to-Prosit-to-DIA approach (*C*). The difference between the predicted (RT =f(iRT)) and the mean measured RT in the DIA data calculated for peptides identified in the DDA-to-DIA (*D*) and in the DDA-to-Prosit-to-DIA (*E*). The overlap between peptides identified in the two approaches is represented in a Venn diagram (*F*). The fraction of peptides predicted as HLA binders are provided for unique and common peptide groups. Nine mer peptides from each group were clustered to reveal the binding motifs. Frequency plot of the delta apex, which is the difference between the measured peptide apex obtained in the DDA-to-DIA and the DDA-to-Prosit-to-DIA (*G*). DDA, data-dependent MS/MS acquisition; DIA, data-independent acquisition; HLA, human leukocyte antigen; MS/MS, tandem MS; RT, retention time.

Recent developments in the application of deep learning algorithms for prediction of MS/MS spectra for a given sequence, collision energy, and charge, offer new possibilities for neoantigen discovery through interrogation of DIA data. Here, we assessed if Prosit (54), a publicly available software based on recent advances in deep learning approaches applied for *in silico* prediction of MS/MS spectra, can be combined with DIA analysis for HLA peptide detection. We generated a library by predicting with Prosit all the HLA peptides included in the BigLib (DDA-to-Prosit-to-DIA). We interrogated the DIA data of RA957, OD5P, and JY samples with this predicted BigLib and compared the results to those obtained initially with the BigLib (DDA-to-DIA) (Fig. 5*A*). First, we inspected the correlations between measured RT and the predicted RT calculated by Spectronaut (RT = f(iRT)) of peptides identified by the two approaches (Fig. 5, *B* and *C* and supplemental Fig. S4). The difference between the predicted RT and the mean measured RT calculated for peptides identified in the DDA-to-DIA analyses was smaller than in the DDA-to-Prosit-to-DIA in all the 3 cell lines (Fig. 5, *D* and *E* and supplemental Fig. S5). In addition, we consistently found a drop of 7 to 10% in the number of peptides identified in the DDA-to-Prosit-to-DIA approach (Fig. 5*F* and supplemental Fig. S5). Nevertheless, the two approaches resulted in similarly accurate identifications, in total 95.7%, 94.4%, and 92.8% of the peptides were predicted as binders in the DDA-to-DIA compared with 96.1%, 95.5%, and 94.7% in the DDA-to-Prosit-to-DIA in OD5P, RA957, and JY cells, respectively. 88.3%, 91.5%, and 89.4% of the peptides identified in the DDA-to-Prosit-to-DIA were also identified in the DDA-to-DIA approach in OD5P, RA957, and JY cells, respectively. In this common set of peptides, 98.3%, 96.8%, and 96.6% were predicted as HLA binders. Among the peptides uniquely detected in each of the approaches, about 75.6% to 82.7% of the peptides were predicted as binders. Clustering the peptides in each of the groups revealed the expected binding motifs (Fig. 5*F* and supplemental Fig. S5). Importantly, among the common peptides, the delta apex of 90% of the PSMs, which is the difference in the DIA-measured peptide apex between DDA-to-DIA and the DDA-to-Prosit-to-DIA approaches, was within 18 s, indicating that both approaches identified the same elution profiles (Fig. 5*G* and supplemental Fig. S5). From this comparison, we concluded that with the current settings, the Prosit-to-DIA approach is as accurate as the DDA-to-DIA; however, more peptides can be identified with the later approach.

**Fig. 6:**
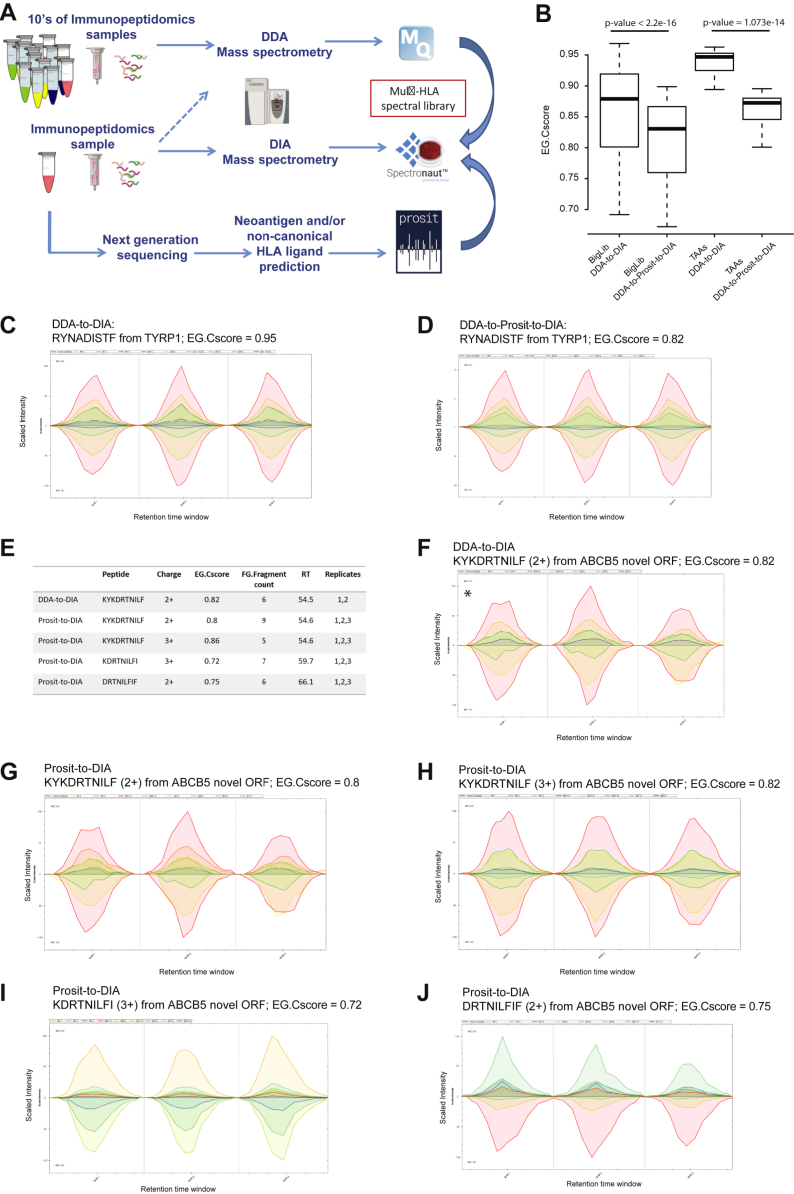
**Prosit-predicted MS/MS spectra for the identification of canonical and noncanonical peptides.** A schematic overview of the application of sample-specific or multi-HLA library for DIA immunopeptidomics in combination with libraries generated from Prosit-predicted MS/MS spectra of canonical and noncanonical peptides (*A*). EG.Cscore distribution of all OD5P peptides and of the 39 TAAs detected in DIA analyzed with the BigLib library (DDA-to-DIA) and the predicted BigLib (DDA-to-Prosit-to-DIA) (*B*). Examples of extracted ion chromatograms for the peptide RYNADISTF detected in the DDA-to-DIA (*C*) and the DDA-to-Prosit-to-DIA (*D*) approaches. Summary of the identified HLA-I peptides form the novel ORF of the ABCB5 gene (*E*). Extracted ion chromatograms of the ABCB5 KYKDRTNILF peptide that have been detected with the DDA-to-DIA (*F*) and with the Prosit-to-DIA approach as doubly (*G*) and triply (*H*) charged ions. Extracted ion chromatograms of the ABCB5+ KDRTNILFI (*I*) and DRTNILFIF (*J*). Extracted ion chromatograms for precursors (MS1) and fragments (MS2) are aligned for each peptide and are shown across the technical replicates. The *asterisk* indicates a manual extraction of transitions that did not pass the q value threshold. DDA, data-dependent MS/MS acquisition; DIA, data-independent acquisition; HLA, human leukocyte antigen; MS/MS, tandem MS; TAAs, tumor-associated antigens.

Next, as a proof of concept, we assessed if clinically relevant antigens could be identified in DIA immunopeptidomics data (Fig. 6*A*) by applying Prosit-based MS/MS prediction. First, we focused on 39 HLA-I peptides derived from TAAs that we have previously identified by Chong *et al.* (20) in the OD5P HLA-I immunopeptidome. We focused on these reference peptides because they have been confirmed by PRM where isotopically heavy-labeled synthetic peptide counterparts were spiked into the OD5P peptidomics sample. All the 39 TAA peptides could be reidentified in the OD5P-DIA samples with the BigLib (DDA-to-DIA) as well as with the Prosit-predicted BigLib (DDA-to-Prosit-to-DIA approaches), with mean EG.Cscores of 0.93 and 0.86, respectively (Welch's two-sample *t* test *p*-value = 1.073e-14), which followed the trend of all identified peptides (Fig. 6*B*, supplemental Tables S7 and S8). For example, the immunogenic HLA-I peptide RYNADISTF from the tyrosinase-related protein 1 was among these TAAs (Fig. 6, *C* and *D*) (20).

Next, we tested if Prosit prediction combined with DIA analysis would allow detection of peptides that failed to be detected by DDA and for which no experimental MS/MS is available. Here, we focused on the detection of noncanonical peptides derived from a novel ORF in the ABCB5 melanoma marker, from which we recently identified one peptide in the OD5P DDA immunopeptidome that was confirmed to be immunogenic (20). We suspected that additional peptides could potentially be presented and detected in the acquired OD5P DIA data. First, we confirmed that also in the DIA data, the immunogenic peptide KYKDRTNILF was detected as a doubly charged ion in two of the three replicates (DDA-to-DIA) (Fig. 6, *E* and *F* and supplemental Table S9). Manual extraction of the ion chromatogram in the third replicate confirmed its detection, yet this PSM did not pass the applied FDR. Then, we created a generic library of predicted MS/MS spectra (Prosit-to-DIA) of 17 9- and 10-mer HLA-I peptides from the ABCB5 translation product predicted to bind frequent HLA allotypes with the PRIME algorithm (55) (supplemental Table S10). With this library, we identified again the KYKDRTNILF peptide, this time as doubly and triply charged ions and in all three replicates (Fig. 6, *G* and *H*). In addition, two new peptides, KDRTNILFI and DRTNILFIF, partially overlapping with the KYKDRTNILF peptide, were identified in all three replicates (Fig. 6, *I* and *J* and supplemental Table S11). We could not reproducibly detect any peptides derived from the ABCB5 novel ORF in the negative controls, JY and RA957 samples (supplemental Table S11). For translational research, and if sufficient amount of sample is available, PRM analyses would provide definite validation for such identifications. We concluded that the integration of Prosit-based prediction of MS/MS spectra with DIA-based immunopeptidomics has great potential in detecting clinically relevant antigens at a higher sensitivity.

## Discussion

In DDA–MS, the stochastic nature of the precursor ion selection in the survey scan before fragmentation leads to low reproducibility across replicates, and often, low abundant ions are not selected for fragmentation. In immunopeptidomics, this lack of consistency results in a large number of missing values in experiments involving multiple samples which significantly impacts the level of reproducibility necessary for assessing differential HLA presentation. In addition, the repertoire of HLA peptides can vary significantly after perturbation in expression of proteins involved in antigen processing and presentation; also, the peptide purification protocol may bias the detectable peptide repertoire. Furthermore, a large fraction of peptides in the immunopeptidome samples are either not selected for fragmentation, they may be masked by other abundant ions, or their identification cannot pass the strict FDR threshold of 1% because of poor fragmentation. However, these peptides might be identified accurately in other samples. On the other hand, a major strength of DIA–MS is the exceptional reproducibility of peptide identification and quantification across multiple experiments. It relies on the availability of peptide MS/MS spectrum libraries, and generating large spectral libraries of HLA-I peptides accurately identified across many samples could be advantageous for sensitive DIA immunopeptidomics. DIA–MS also generates permanent quantitative digital maps and enables highly reproducible retrospective analysis and mining of HLA data.

In proteomics, false identifications in DIA analyses are often tested by mixing up samples of similar complexity but of different origin, such as yeast and human, and by matching them against a library comprising only one of the sources (51). In immunopeptidomics, to some extent, the samples are mixed by nature, as they comprise different HLA allotypes and binding motifs. Therefore, in this study, newly generated immunopeptidomics DIA data of three different samples were independently matched against spectral libraries of growing complexity, and we monitored the fraction of peptides that are predicted to bind the respective HLA allotypes and clustered the identified peptides to reveal the binding specificities to systematically assess its accuracy. A high level of false identifications would have been visible as interfering motifs that are not authentic to the HLA allotypes expressed in the investigated sample and by a drop in the percentage of peptides predicted as binders. Our results obtained from the application of sample-specific libraries confirmed the anticipated enhanced sensitivity. The specificity was comparable with the DDA approach. Furthermore, results from the combined, mixed, and the BigLib multi-HLA libraries suggested no, or minimal, propagation of false identifications. As each HLA allotype is characterized by a slightly different peptide length preference (47), small variations are expected between DDA and DIA data, when matched against multi-HLA library, as the coverage of peptide identifications is not equally distributed among the expressed allotypes and is dependent on the breadth of peptides available in the library. We concluded that the fraction of predicted binders is an informative and unique feature of HLA peptides that performed well for assessing accuracy of DIA immunopeptidomics in the context of a multi-HLA library such as the BigLib.

Several limitations are anticipated for this approach. Here, we tested the feasibility of matching DIA immunopeptidomics measurements against libraries constructed from DDA measurements that were acquired from the exact same biological sample, different biological replicates that were measured sequentially, or DDA measurements of variety of samples measured several months apart, yet, with comparable LC-MS/MS settings. Therefore, no iRT internal standards were included. Our results indicate that research groups may use their internal archive of DDA immunopeptidomics measurements, which were prepared and measured with same LC-MS/MS protocols, for the creation of multiallelic libraries for their internal use. In the future, a larger study would be required to overcome interlab variability when different LC gradients are applied. Inclusion of iRT internal standards would be vital to enable inclusion of historical data from different laboratories and data generated from newer or upgraded LC-MS/MS instrumentations (57). In addition, other endogenous peptides that are typically well detected in a given sample could be used to calibrate RT (58).

In addition, when permissive FDR is set for the DDA data analysis used for the construction of the library, errors would be propagated to the identifications of the DIA-derived peptides. As such wrong identifications are rather random, it would be challenging to monitor their prevalence. Hence, it is recommended to apply stringent FDR thresholds at the library construction step. Additional errors can be introduced at the level of DIA spectral matching. If the library contains only peptide sequences of the expected HLA-binding specificities according to the expressed HLA allotypes in the investigated sample, as reported previously (39), monitoring the level of error would again be challenging. However, if the library contains an excess of competing peptides of unrelated HLA-binding specificities, the level of false matching and identifications could be estimated by the elevated level of peptides that are unlikely to bind the expressed HLA alleles.

From this study, we concluded that a comprehensive multi-HLA library for the DIA approach would be highly advantageous in clinical settings where often only a low amount of biological sample is available for immunopeptidomics. We mimicked this scenario by generating minimal sample-specific libraries from a few DDA measurements that were, as expected, not comprehensive enough to yield high identification rates. When samples express frequent HLA alleles that are covered in the multi-HLA library, or HLA alleles with degenerate motifs, a large number of peptides can be identified even when no sample-specific DDA data are included in the library. Furthermore, we showed that samples diluted 5-fold reached a high sensitivity that is comparable with the undiluted samples. Hence, with a comprehensive multi-HLA library, the breadth of the immunopeptidome could be captured by DIA also in case of sparse clinical samples that express HLA allotypes characterized with common binding motifs. Of note, we observed a decrease in detection of around 500 peptides matching DIA data of OD5P against the OD5P+RA957 library, compared with the coverage obtained with the OD5P-specific library. FDR calculations applied to different combinations of samples could slightly alter the peptide yields and the repertoire, for example, at the MaxQuant level. However, we consistently found an increase in coverage using the larger mixed and BigLib libraries. Very rarely, all six motifs of HLA class I molecules expressed in a given sample are rare or exceptional; therefore, we anticipate that almost every sample will benefit from this approach, yet to a different extent. When samples express HLA allotypes that are not yet sufficiently covered in the library, additional sample-specific DDA data would provide adequate depth and would increase the coverage of the library for future applications. If the sample amount is not the limiting factor, multiple DDA immunopeptidomics measurements of samples extracted with different affinity purification protocols and with extensive peptide fractionation, combined with sample-specific optimization of the DIA parameters (*e.g.*, precursor mass range, width of the isolation windows, and the collision energy), are expected to increase the sensitivity of the DIA analysis (40, 59, 60).

Currently, the development of DDA-independent approaches for DIA data analysis is rapidly advancing (54). We have shown here, as a proof of concept, that the integration of Prosit-based prediction of MS/MS spectra with DIA-based immunopeptidomics has a great potential for sensitive detection of mutated neoantigens and other canonical and noncanonical HLA ligands that could be clinically relevant. Shortlisting targets, such as mutated neoantigens, based on evidence of detection at the immunopeptidome, has been shown to be effective in enriching the target list with peptides capable of rejecting tumors in vaccination studies in mouse models (61) or that can be immunogenic in humans (15). Therefore, for the development of exploratory neoantigen-based therapies, when no archived samples are available for comprehensive targeted-MS analyses or when the time allocated for target identification is limited, it is reasonable to prioritize the high-confidence hits based on the collective evidences of expression and HLA presentation. Our data suggest that Prosit-like MS/MS spectrum predictors could pave the way for massive and effortless assessment of presentation-predicted HLA ligands. Yet, some parameters should be adapted specifically for immunopeptidomics, such as the flexibility of carbamidomethylation on cysteine, which is frequent in proteomics, but only a few peptides are detected with this modification in immunopeptidomics. Alternatively, adding an alkylation step to the protocol of HLA peptide purification could potentially overcome this limitation. Training such predictors with large-scale datasets of purified HLA peptides or similar synthetic counterparts will help improve the accuracy and the performance of this approach.

In recent years, DDA-independent approaches for DIA data analyses have been greatly improved (62, 63) and are expected to be more straightforward than a DDA-dependent DIA approach; however, these have not yet been tested or optimized for nontryptic immunopeptidomics data, and the FDR calculation due to the large space search should be carefully assessed. Nevertheless, large-scale immunopeptidomics datasets are publicly available (64, 65), and many immunopeptidomics laboratories have immense archived data, each typically measured with similar LC-MS settings. These may be suitable for the construction of comprehensive multi-HLA spectral libraries that could facilitate highly sensitive DIA immunopeptidomics. This approach is expected to advance the implementation of immunopeptidomics in basic and translational research domains where samples with limited amounts of biological material are to be interrogated.

## Data Availability

Newly generated MS raw files and all MaxQuant and Spectronaut output results have been deposited to the ProteomeXchange Consortium *via* the PRIDE (43) partner repository with the dataset identifier PXD022950.

## Supplemental data

This article contains supplemental data.

## Conflict of interest

The authors declare no competing interests.
