# Supplementary material for: Sensitive Immunopeptidomics by Leveraging Available Large-Scale Multi-HLA Spectral Libraries, Data-Independent Acquisition, and MS/MS Prediction
Source: Mol Cell Proteomics. 2021 Apr 9;20:100080. doi: 10.1016/j.mcpro.2021.100080 (PMC8724634; doi:10.1016/j.mcpro.2021.100080)
Supplement: Supplemental information [file mmc12.docx]

Supplemental information

**Sensitive immunopeptidomics by leveraging available large-scale multi-HLA spectral libraries, data-independent acquisition and MS/MS prediction**

HuiSong Pak^1^, Justine Michaux^1^, Florian Huber^1^, Chloe Chong^1^, Brian J. Stevenson^2^, Markus Müller^1,2^, George Coukos^1^, Michal Bassani-Sternberg^1^

^1^Department of Oncology, Ludwig Institute for Cancer Research Lausanne, Lausanne University

Hospital and the University of Lausanne, Lausanne, Switzerland.

^2^SIB Swiss Institute of Bioinformatics, Lausanne, Switzerland

**Corresponding author:** Michal Bassani-Sternberg Michal.bassani@chuv.ch

**Supplemental Table 1:** A list of all DIA and DDA raw files that were included in this study, the source of samples and PRIDE identifiers related to previously published data.

**Supplemental Table 2:** High resolution HLA typing information for all samples included in this study. **Supplemental Table 3:** Description of the windows of MS/MS acquisitions. For each DIA MS/MS scan, a resolution of 30,000, an AGC of 3e6 and a ramping normalized collision energy (NCE = 25.5, 27 and 30) were used.

**Supplemental Table 4:** Spectronaut output of identified peptides in each of the JY, 0D5P, and RA957 DIA samples matched against the different sample-specific libraries. Columns corresponding to PG.Genes, PG.UNiProtIds, PG.ProteinNames, PEP.StrippedSequence, EG.PrecursorId, EG.ModifiedSequence, PEP.Quantity, EG.Qvalue and EG.ApexRT are provided.

**Supplemental Table 5:** Spectronaut output of identified peptides in each of the JY, 0D5P, and RA957 DIA samples matched against the different combined libraries. Columns corresponding to PG.Genes, PG.UNiProtIds, PG.ProteinNames, PEP.StrippedSequence, EG.PrecursorId, EG.ModifiedSequence,

PEP.Quantity, EG.Qvalue and EG.ApexRT are provided.

**Supplemental Table 6:** Spectronaut output of identified peptides in each of the JY, 0D5P, and RA957 DIA samples matched against the mixed library. Columns corresponding to PG.Genes, PG.UNiProtIds,

PG.ProteinNames, PEP.StrippedSequence, EG.PrecursorId, EG.ModifiedSequence, PEP.Quantity, EG.Qvalue and EG.ApexRT are provided.

**Supplemental Table 7:** Spectronaut output of identified peptides in each of the JY, 0D5P, and RA957

DIA samples matched against the BigLib library. Columns corresponding to PG.Genes, PG.UNiProtIds, PG.ProteinNames, PEP.StrippedSequence, EG.PrecursorId, EG.ModifiedSequence, PEP.Quantity, EG.Qvalue and EG.ApexRT are provided.

**Supplemental Table 8:** Spectronaut output of identified peptides in each of the JY, 0D5P, and RA957

DIA samples matched against the Prosit-predicted BigLib library (DDA-to-Prosit-to-DIA). Columns corresponding to PG.Genes, PG.UNiProtIds, PG.ProteinNames, PEP.StrippedSequence, EG.PrecursorId, EG.ModifiedSequence, PEP.Quantity, EG.Qvalue and EG.ApexRT are provided.

**Supplemental Table 9:** Spectronaut output of identified peptides in each of the JY, 0D5P, and RA957

DIA samples matched against the Uniprot+ABCB5 library (DDA-to-DIA). Columns corresponding to PG.Genes, PG.UNiProtIds, PG.ProteinNames, PEP.StrippedSequence, EG.PrecursorId, EG.ModifiedSequence, PEP.Quantity, EG.Qvalue and EG.ApexRT are provided.

**Supplemental Table 10:** A generic list of 9- and 10-mer HLA-I peptides from the ABCB5 novel ORF predicted to bind frequent HLA allotypes (A*01:01, A*02:01, A*03:01, A*24:02, A*26:01, B*07:02, B*08:01, B*27:05, B*39:01, B*40:01, B*58:01, B*15:01) generated with the PRIME (https://github.com/GfellerLab/PRIME) algorithm (rank ≤1%).

**Supplemental Table 11:** Spectronaut output of identified peptides in each of the JY, 0D5P, and RA957

DIA samples matched against the Prosit predicted ABCB5 library (Prosit-to-DIA). Columns corresponding to PG.Genes, PG.UNiProtIds, PG.ProteinNames, PEP.StrippedSequence, EG.PrecursorId, EG.ModifiedSequence, PEP.Quantity, EG.Qvalue and EG.ApexRT are provided.
