# Supplementary material for: Sensitive Immunopeptidomics by Leveraging Available Large-Scale Multi-HLA Spectral Libraries, Data-Independent Acquisition, and MS/MS Prediction
Source: Mol Cell Proteomics. 2021 Apr 9;20:100080. doi: 10.1016/j.mcpro.2021.100080 (PMC8724634; doi:10.1016/j.mcpro.2021.100080)
Supplement: Supplemental Figure S1 [file mmc13.pdf]

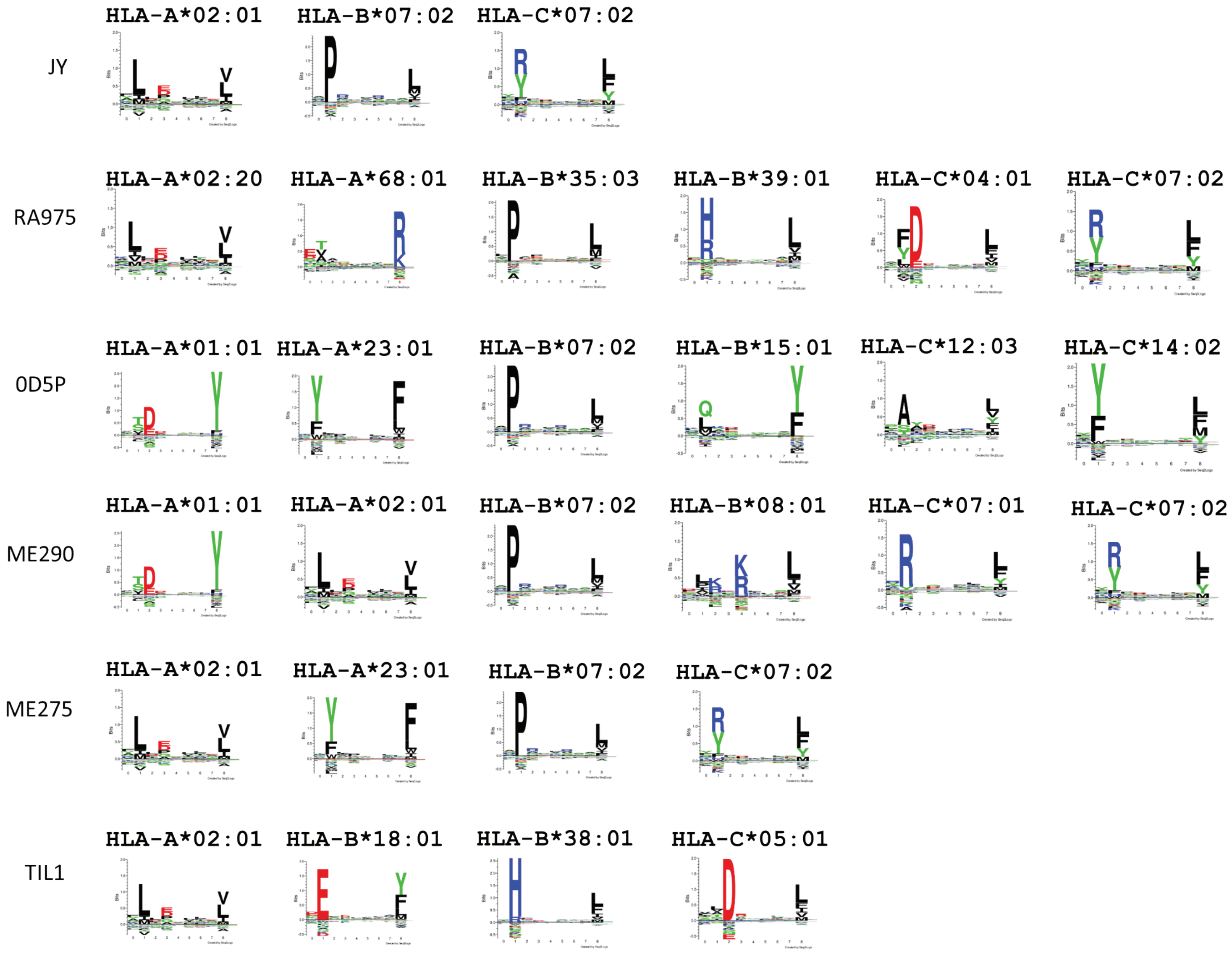

Supplemental Figure 1. Reference HLA binding motifs from NetMHCpan motif viewer of HLA allotypes expressed in the six selected samples that were analyzed in this study and that were included in the sample-specific, combined and mixed libraries.
