# Supplementary material for: Sensitive Immunopeptidomics by Leveraging Available Large-Scale Multi-HLA Spectral Libraries, Data-Independent Acquisition, and MS/MS Prediction
Source: Mol Cell Proteomics. 2021 Apr 9;20:100080. doi: 10.1016/j.mcpro.2021.100080 (PMC8724634; doi:10.1016/j.mcpro.2021.100080)
Supplement: Supplemental Figure S2 [file mmc14.pdf]

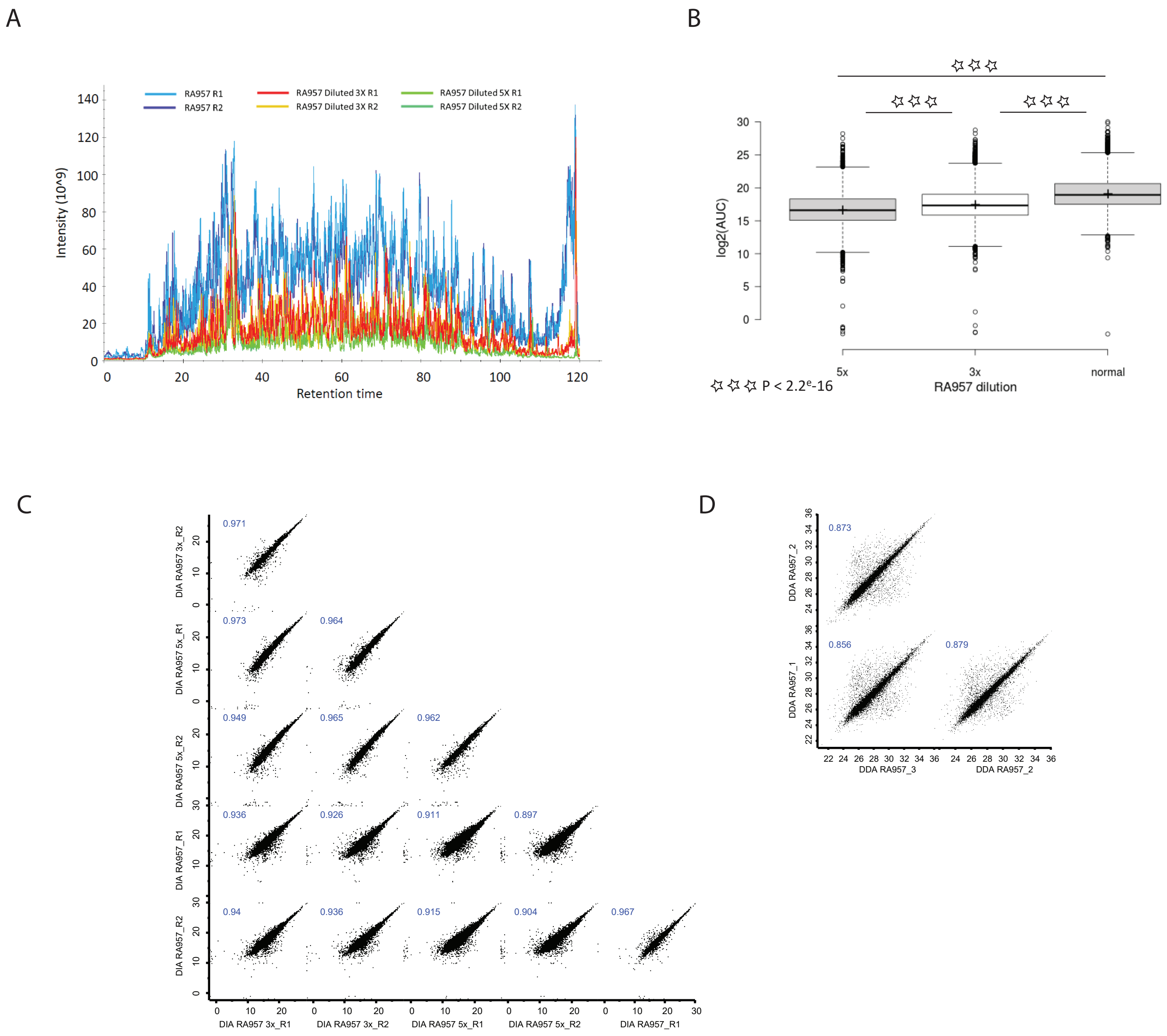

Supplemental Figure 2: (A) Total ion chromatogram (TIC) intensities for each of the replicates of RA957 (normal, 3x and 5x diluted) DIA measurements is provided. **(B)** Comparison of averaged Log2(AUC) values of all identified peptides in RA957 normal, 3x and 5x diluted DIA replicates without global intensity normalization. Quantitative reproducibility of the RA957 immunopeptidomes measured by DIA **(C)** and by DDA **(D)** without global intensity normalization. Pearson Correlation values (r) are provided.
