# Supplementary material for: Sensitive Immunopeptidomics by Leveraging Available Large-Scale Multi-HLA Spectral Libraries, Data-Independent Acquisition, and MS/MS Prediction
Source: Mol Cell Proteomics. 2021 Apr 9;20:100080. doi: 10.1016/j.mcpro.2021.100080 (PMC8724634; doi:10.1016/j.mcpro.2021.100080)
Supplement: Supplemental Figure S3 [file mmc15.pdf]

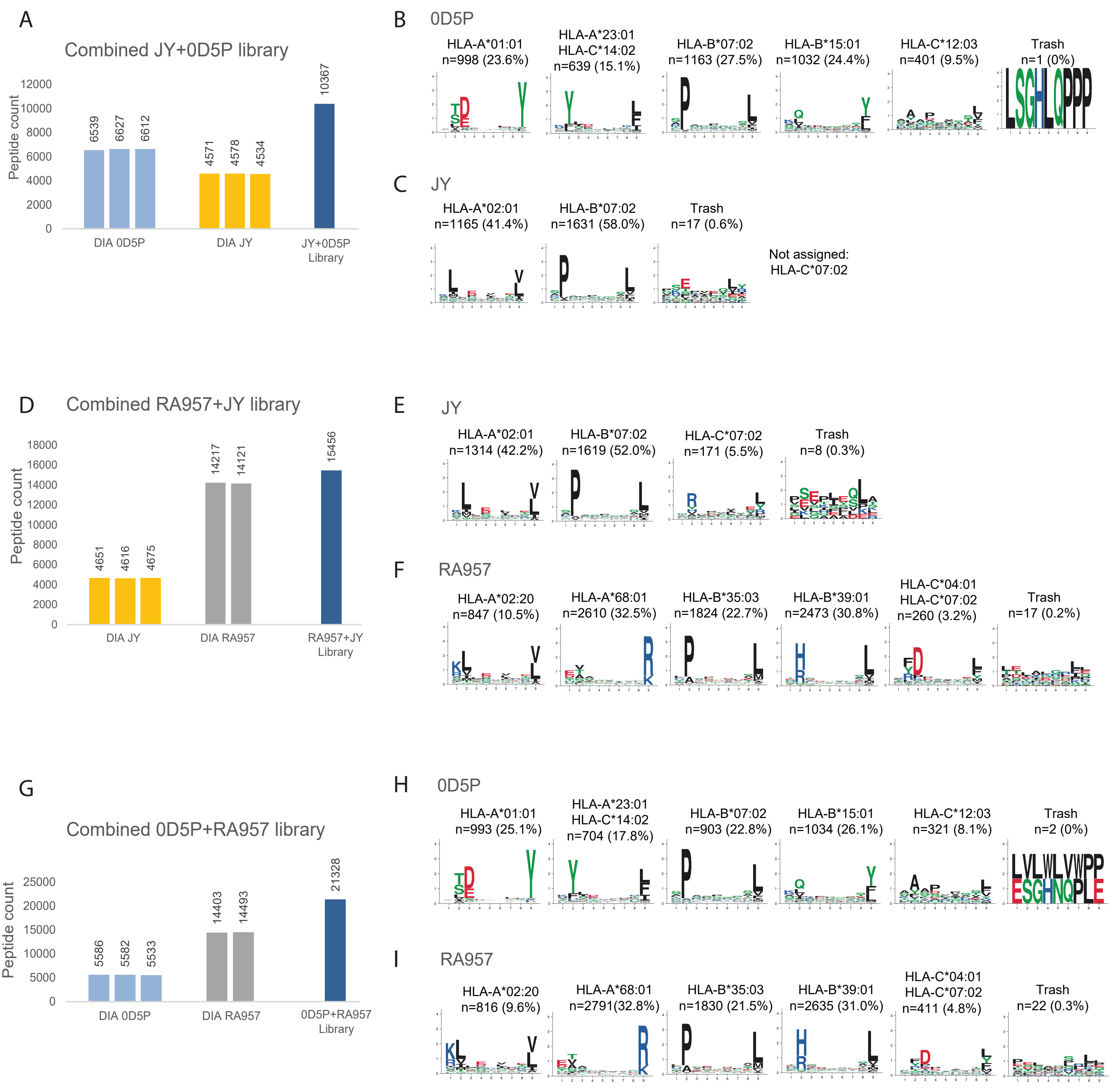

Supplemental Figure 3: Application of combined spectral libraries for matching immunopeptidomics DIA data. **(A)** DIA data of JY and 0D5P samples were matched against the JY+0D5P combined library. The number of peptides identified in each of the DIA measurements and the number of peptides included in the JY+0D5P library are reported. Deconvolution of the consensus binding motifs of 0D5P **(B)** and JY **(C)** DIA immunopeptidomics samples with MixMHCp 2.1 and manual annotation of motifs. The number of peptides and the HLA restriction assigned to each motif are reported. **(D)** DIA data of RA957 and JY samples were matched against the RA957+JY combined library. The number of identified peptides is provided like in A. Deconvolution of the consensus binding motifs of JY **(E)** and RA957 **(F)** DIA immunopeptidomics samples. **(G)** DIA data of 0D5P and RA957 samples were matched against the 0D5P+RA957 combined library. The number of identified peptides is provided like in A. Deconvolution of the consensus binding motifs of 0D5P **(H)** and RA957 **(I)** DIA immunopeptidomics samples.
