# Supplementary figures and images for: Sensitive Immunopeptidomics by Leveraging Available Large-Scale Multi-HLA Spectral Libraries, Data-Independent Acquisition, and MS/MS Prediction
Source: Mol Cell Proteomics. 2021 Apr 9;20:100080. doi: 10.1016/j.mcpro.2021.100080 (PMC8724634; doi:10.1016/j.mcpro.2021.100080)

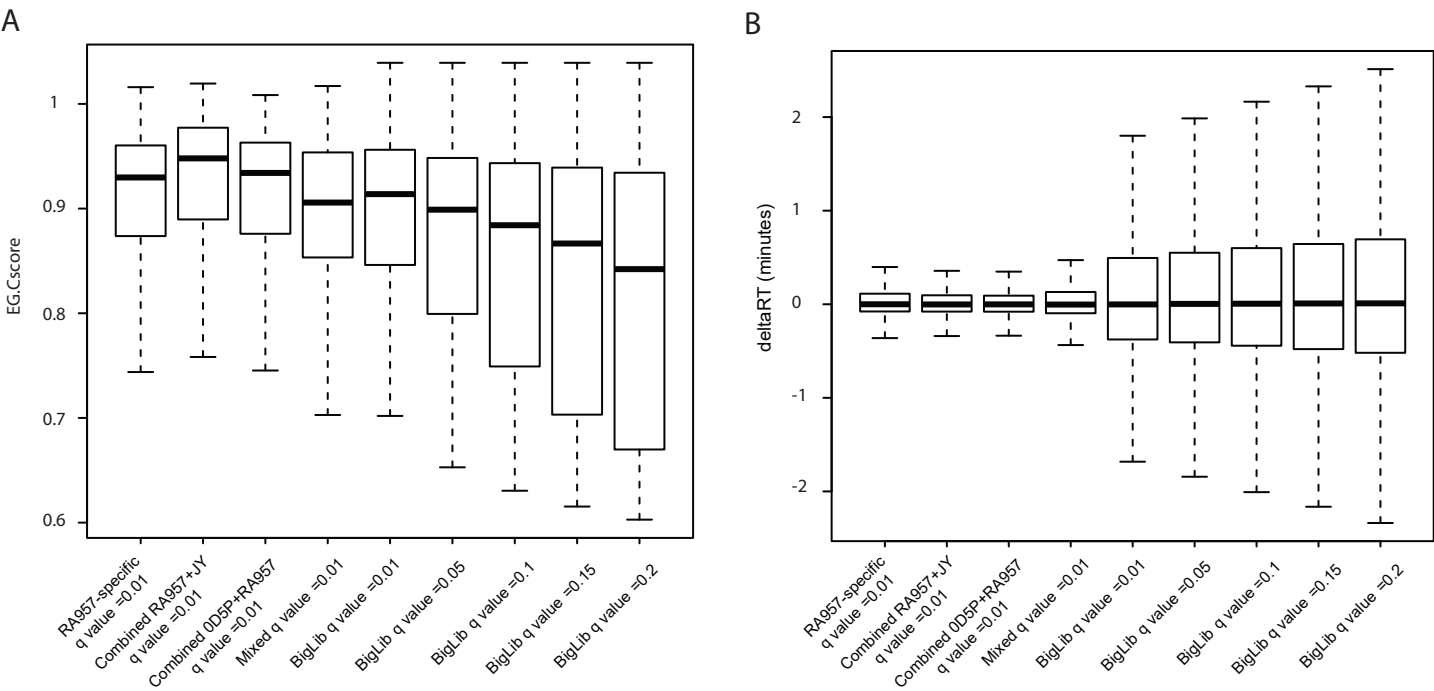

Supplement: Supplemental Figure S4 [file mmc16.pdf]
