# Supplementary material for: Sensitive Immunopeptidomics by Leveraging Available Large-Scale Multi-HLA Spectral Libraries, Data-Independent Acquisition, and MS/MS Prediction
Source: Mol Cell Proteomics. 2021 Apr 9;20:100080. doi: 10.1016/j.mcpro.2021.100080 (PMC8724634; doi:10.1016/j.mcpro.2021.100080)
Supplement: Supplemental Figure S5 [file mmc17.pdf]

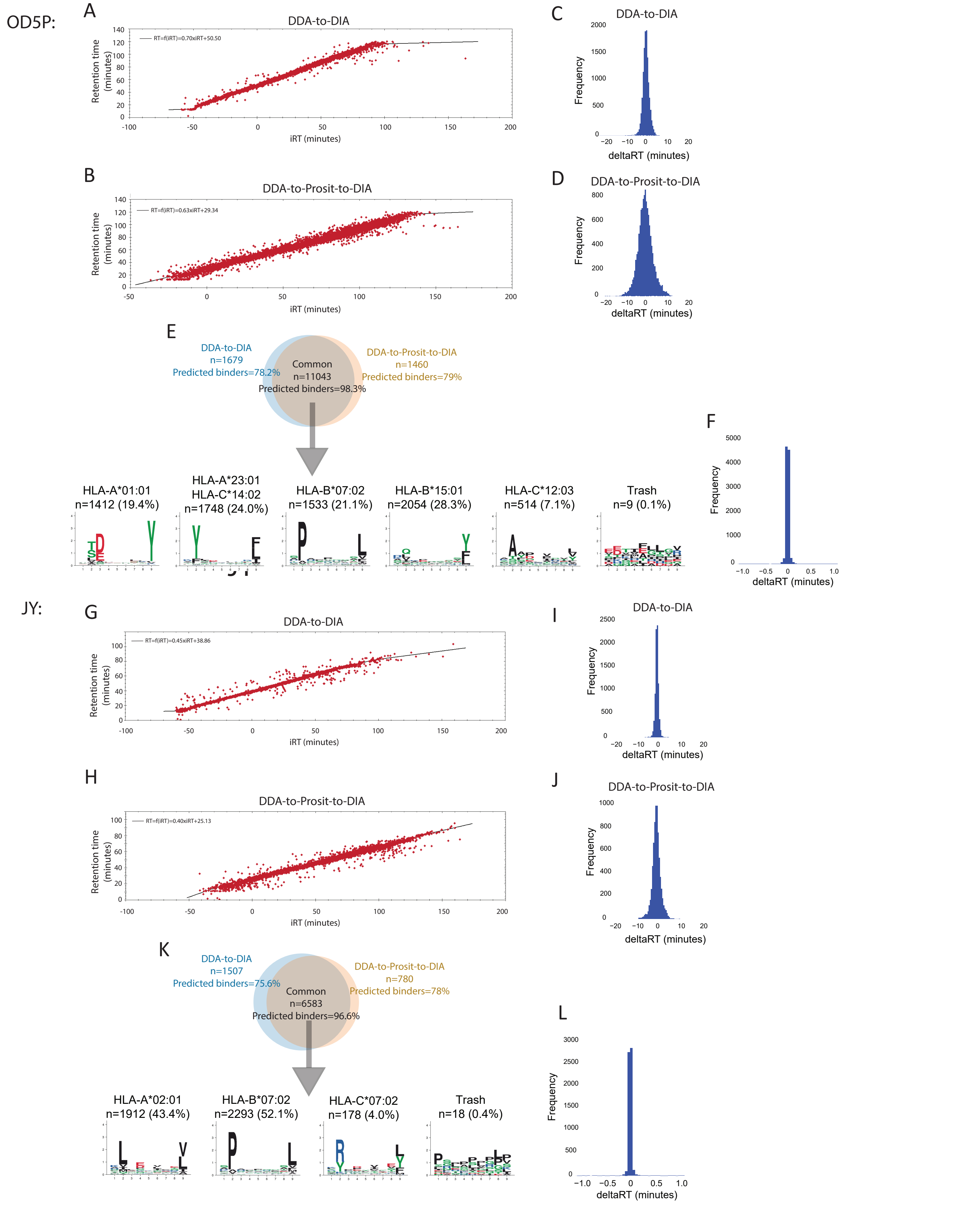

Supplemental Figure 5: Correlations between measured RT and the predicted RT calculated by Spectronaut (RT = f(iRT)) of peptides identified in OD5P samples applying the BigLib library in the DDA-to-DIA approach (**A**) and with the Prosit-predicted BigLib library in the DDA-to-Prosit-to-DIA approach (**B**). The difference between the predicted RT =f(iRT)) and the mean measured RT in the DIA data calculated for peptides identified in the DDA-to-DIA (**C**) and in the DDA-to-Prosit-to-DIA (**D**). The overlap between peptides identified in the two approaches is represented in a venn diagram (**E**). The fraction of peptides predicted as HLA binders is provided for unique and common peptide groups. Common 9 mer peptides were clustered to reveal the binding motifs. Frequency plot of the delta apex, which is the difference between the measured peptide apex obtained in the DDA-to-DIA and the DDA-to-Prosit-to-DIA (**F**). The same analyses as above are provided for JY samples (**G-L**).
